# A Single Dose of *Ginkgo biloba* Extract Induces Gene Expression of Hypothalamic Anorexigenic Effectors in Male Rats

**DOI:** 10.3390/brainsci11121602

**Published:** 2021-12-02

**Authors:** Meira M. F. Machado, Janilda P. Pereira, Bruna K. S. Hirata, Viviane S. Júlio, Renata M. Banin, Heider M. Andrade, Eliane B. Ribeiro, Suzete M. Cerutti, Mônica M. Telles

**Affiliations:** 1 Post-Graduate Program in Chemical Biology, Institute of Environmental Sciences, Chemical and Pharmaceutical, Universidade Federal de São Paulo, Diadema 09972-279, Brazil; meyramachado@gmail.com (M.M.F.M.); jannilda.pereira@gmail.com (J.P.P.); bruhirata@gmail.com (B.K.S.H.); viviane_julio@hotmail.com (V.S.J.); heider07@gmail.com (H.M.A.); smcerutti@unifesp.br (S.M.C.); 2 Discipline of Nutrition Physiology, Department of Physiology, Universidade Federal de São Paulo, Botucatu Street-Edifício de Ciências Biomédicas, 2º Andar–Vila Clementino, São Paulo 04021-001, Brazil; renatamancini.banin@gmail.com (R.M.B.); eliane.beraldi@unifesp.br (E.B.R.)

**Keywords:** food intake GbE, hypothalamic neuropeptides, serotonergic system

## Abstract

Previous studies have shown that *Ginkgo biloba* extract (GbE) reduces food intake and body mass gain and regulates proteins related to lipid metabolism in obese rats. In ovariectomized rats, GbE restored the hippocampal and hypothalamic serotonergic system activity, favoring the spontaneous feeding decrement. Considering the promising hypophagic effect of GbE, this study aimed to investigate the effect of a single acute dose on hypothalamic pathways that regulate feeding behavior in male rats. Four-month-old Wistar male rats received either a single acute oral GbE dose (500 mg/kg) or vehicle. Food intake and body mass were measured after 1, 4, 12, and 24 h. Rats were euthanized, and hypothalami were removed for mRNA quantification of anorexigenic (POMC/CART) and orexigenic (AgRP/NPY) neuropeptides, leptin/serotonin receptors (5HT1A, 5HT1B, 5HT2C), and serotonin transporters. We also investigated POMC, 5-HT1B, and 5-HT2C protein levels. A single acute GbE dose induced the hypothalamic POMC, CART, and 5-HT2C gene expression but failed to modify orexigenic effectors. No alterations in food intake, body mass, and hypothalamic protein levels were observed. In summary, the present findings demonstrate the rapid stimulation of pivotal hypothalamic anorexigenic pathways in response to a single GbE administration, reinforcing the GbE hypophagic activity. However, more studies are necessary to evaluate its potential as an appetite modulator.

## 1. Introduction

The regulation of energy homeostasis and feeding behavior are mainly performed by the hypothalamus, a central area responsible for the integration of peripheral and neural signals regarding feeding and body energy status [1]. Therefore, the hypothalamus coordinates responses, stimulating or inhibiting the appetite, through the action of orexigenic and anorexigenic pathways, respectively [2]. Some important hypothalamic mediators of energy homeostasis are the neuropeptide Y (NPY) and agouti-related peptide (AgRP) orexigenic effectors, while the proopiomelanocortin (POMC) and cocaine and amphetamine-regulated transcript (CART) are recognized as pivotal anorexigenic effectors [3]. Moreover, peripheral hormones, such as the adipokine leptin, and the pancreatic hormone insulin inform the hypothalamus about the long-term energy status to reduce the activity of orexigenic pathways and stimulate the anorexigenic signaling [1,4,5].

The serotonergic system is a key modulator involved in the control of feeding behavior. It is well established that, during a meal, the extracellular hypothalamic serotonin levels raise in order to promote satiation, and thus interrupting the acute food intake [6,7]. This hypophagic response associated with the serotonin activity is mainly related to the stimulation of POMC/CART neurons by the activation of the 5-HT2C receptor [8]. A similar role for the 5-HT1B receptor was reported, since its activation was demonstrated to suppress food intake in mice, illustrating its important role in satiety promotion [9]. Indeed, Heisler and colleagues [10] demonstrated that the activation of the 5-HT1B receptor inhibited NPY/AgRP neuronal activity, while it reduced the GABAergic signaling onto POMC neurons. On the other hand, both the activation of the hypothalamic 5-HT1A heteroreceptors present on the NPY and AgRP orexigenic neurons [11] and dorsal raphe 5-HT1A somatodendritic auto-receptors [12] have been reported to induce feeding, and thus, the balance on 5-HT receptors activation is crucial to the energy homeostasis maintenance.

Due to the complex regulation of energy homeostasis, which needs to be performed in a coordinated manner to be maintained, disturbances triggered in components involved in the generation of these responses may contribute to the expansion of body adiposity and favor the appearance of obesity-related comorbidities [1,5].

Considering that many drugs broadly commercialized as appetite suppressors and body mass controllers are associated with all sorts of side effects, as well as with their potential for addiction, the study of new therapeutic approaches able to modulate energy homeostasis and feeding behavior without adverse events is extremely necessary [13]. In this scenario, we have demonstrated that the standardized *Ginkgo biloba* extract (GbE), one of the most commercialized supplements worldwide [14], presents promising anti-obesogenic properties. Indeed, we have previously reported that GbE supplementation for 14 days reduced food intake, body mass gain, and visceral adiposity in diet-induced obese male rats (DIO) [15,16,17]. Moreover, GbE improved insulin signaling in both the retroperitoneal adipose tissue and gastrocnemius muscle [15,16], as well as modulated proteins involved in lipid metabolism [17,18], and attenuated the inflammation and oxidative stress of retroperitoneal fat depot in obese rats [17,18].

In ovariectomized rats (OVX), GbE also decreased food intake and improved the body composition and lipid profile, which might have been resulted, at least in part, from the stimulation of the serotonergic system, since the GbE supplementation improved the serotonin levels in the ventromedial hypothalamus (VMH) [19,20]. In addition, the modulation of serotonergic system activity in the hippocampus through the re-establishment of 5-HT1A and 5-HT1B receptor levels [21] might be also involved in the improvement of anxious- and depressive-like behaviors observed after GbE supplementation [20]. Additionally, the GbE supplementation exerted an important modulatory effect on leptinergic signaling, as observed by the reduction in leptin serum levels as well as the restoration of hippocampal leptin receptors, reinforcing the capacity of GbE to enhance anorexigenic responses [20,21].

Since important anorexigenic and anti-obesogenic effects were observed after a prolonged supplementation with GbE in different animal models, the present study aimed to investigate the initial sequence of events related to the anorexigenic effects of GbE. Thus, it was evaluated if a single acute dose of GbE alters pivotal hypothalamic mediators involved in the modulation of feeding behavior in male rats.

## 2. Materials and Methods

### 2.1. Animals

The Ethics Committee on Animal Research of the Universidade Federal de São Paulo (process number 2690100414) approved all procedures, following the principles of the Brazilian law for the use of animal models in research (Arouca Law—number 11794/08). Throughout the experimental period, the animals were maintained under controlled temperature (23 ± 1 °C) and in light/dark cycle of 12h/12h (lights on from 6 a.m. to 6 p.m.), with free access to water and a balanced diet (Nuvilab^®^, Brazil, 2.79 Kcal.g^−1^).

### 2.2. Experimental Protocol

The *Ginkgo biloba* extract used in the present study was commercialized by Huacheng Biotech Inc. (Changsha, China) and contains flavonoids (25.21%—quercetin, isorhamnetin, kaempferol, and rutin) and terpenoids (6.62%—bilobalide, ginkgolide B and C). Bioactive compounds of the extract used in the present study were identified and published by Machado and colleagues [21]. The amount of 25.1% of flavonoids present in the GbE herein used is acceptable when considering that the main *Ginkgo biloba* extract—EGb 761 (Tebonin^®^, commercialized by Dr. W. Schwabe GmbH, Karlsruhe, Germany)—may contain 22% to 27% of flavonoids content.

For habituation, 4-month-old male Wistar rats were all sham-gavaged with 1 mL saline before the start of the experiment, and then food intake was measured 1, 4, 12, and 24 h after the procedure. For the experimental protocol, animals were separated into two groups: (1) receiving only saline gavage (Control group, *n* = 4–6) or (2) a single oral dose of GbE 500 mg.kg^−1^ diluted in 1 mL of saline (GbE group, *n* = 5–6) [22].

Then, food intake was measured 1, 4, 12, and 24 h after gavage. Food intake was calculated as the difference between the amount of chow offered and the remaining after the periods specified above. Moreover, animals were weighed before and 24 h after the treatment. The dose of GbE herein used was determined based on previous studies [15,16,22,23].

### 2.3. Gene Expression Analysis—Real-Time PCR

Twenty-four hours after gavage with saline or GbE, rats were deeply anesthetized with sodium thiopental (80 mg/kg, i.p.) and euthanized, and then hypothalami were rapidly removed and stored at −80 °C until the use. Posteriorly, the hypothalami were homogenized with Trizol (Invitrogen^®^, Carlsbad, CA, USA), and RNA extraction was performed according to the manufacturer’s instructions. RNA concentration was evaluated at 260/280 nm and 260/230 in Nanodrop spectrophotometer (Thermo Scientific^®^, Loughborough, UK), while the complementary DNA (cDNA) was synthesized with a High-Capacity cDNA kit (Applied Biosystems^®^, Foster City, CA, USA) following the manufacturer’s recommendation. Taqman probes (Life Technologies^®^, Carlsbad, CA, USA) were used for the gene expression analysis of neuropeptides POMC (Rn00595020_m1), CART (Rn01645174_m1), AgRP (Rn01431703_g1), and NPY (Rn01410145_m1). The mRNA expression of serotonin receptors 5-HT1A (Rn00561409_s1), 5-HT1B (Rn01637747_s1), 5-HT2C (Rn00562748_m1), and leptin receptor LEPr (Rn01433205_m1) were also evaluated, as well as serotonin transporter 5-HTT (Rn00564737_m1) and constitutive gene actin β (Rn00667869_m1). Carefully following the recommendations of the manufacturer, the real-time PCR experiments were conducted with one sample from each experimental group, and reactions were carried in three replicates. The results were analyzed using the StepOne software (v.2.3) provided by the manufacturer.

### 2.4. Western Blotting

Hypothalami were homogenized in lysis buffer (complete ULTRA Tablets—Mini Roche^®^; 100 mM Tris; 10 mM EDTA; 10% Triton X-100), and after 30 min, samples were centrifuged at 16.000× *g* for 40 min at 4 °C. The protein concentration was measured by BCA kit (Bio-Rad, Hercules, CA, USA). Proteins were separated on 8% SDS-PAGE and transferred to nitrocellulose membrane (GE Healthcare, Pittsburgh, PA, USA) by a semi-dry transfer cell system (Bio-Rad Hercules, CA, USA). The membranes were blocked in blocking buffer (5% bovine serum albumin—BSA; 1M Tris; 0.02% Tween-20; 5M NaCl) for 2h at 4 °C and incubated overnight at 4 °C with the following primary antibodies: anti-serotonin receptors 5-HT1B (Abcam-ab13896) and 5-HT2C (Abcam-ab133570), as well as anti-POMC neuropeptide (Abcam-ab180766) and β-tubulin (Cell Signaling-2146). The membranes were then incubated with specific horseradish peroxidase–conjugates anti-mouse IgG (Cell signaling-7076S) or anti-rabbit IgG (Cell signaling-7074S) antibodies, with posterior detection by chemiluminescence (ECL reagent, GE Healthcare Biosciences, Pittsburgh, PA, USA). Posteriorly, the proteins were quantified by densitometry using the Scion Image^®^ software (Scion Corporation, Frederick, MD, USA).

### 2.5. Statistical Analysis

Data were expressed as Mean ± Standard Error Mean (SEM). The variables were tested for normality using the Shapiro–Wilk test. Comparisons between the groups were performed using Mann–Whitney or Student’s t-tests. Statistical analyses were performed using Prism version 6.0 (GraphPad Software, Inc., San Diego, CA, USA). The level of significance was set at *p* < 0.05.

## 3. Results

### 3.1. Food Intake and Body Mass Profile

To minimize the associated stress with orogastric gavage, it was handled by a sham gavage before the start of GbE supplementation. Table 1 shows food intake 1, 4, 12, and 24 h after sham saline gavage of both groups and then, 1, 4, 12, and 24 h posterior to GbE supplementation (GbE). Posteriorly to sham saline gavage, it was not observed differences in food intake at 1 h (*p* = 0.161), 4 h (*p* = 0.588), 12 h (*p* = 0.381), and 24 h (*p* = 0.915) subsequently. Similarly, after group division, no differences were detected among the Control and GbE groups at any time point evaluated (1 h—*p* = 0.088; 4 h—*p* = 0.099; 12 h—*p* = 0.192; 24 h—*p* = 0.127). Regarding body mass (Table 2), no statistical differences were identified neither before nor 24 h after the saline or GbE gavages.

### 3.2. Gene Expression of Hypothalamic Neuropeptides Involved in the Central Control of Food Intake

**Figure 1:**
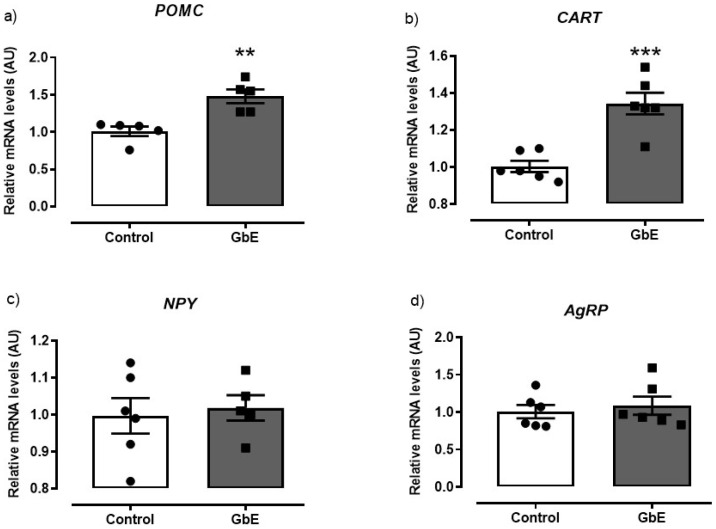
Relative mRNA levels of the hypothalamic neuropeptides: POMC (**a**), CART (**b**), NPY (**c**), and AgRP (**d**) of Control (*n* = 5–6) and *Ginkgo biloba* (GbE; *n* = 5–6) groups analyzed by RT-qPCR. ** *p* < 0.01, *** *p* < 0.001.

The mRNA quantification of anorexigenic neuropeptides demonstrated a significant enhancement after a single acute dose of GbE in comparison to the Control group (Figure 1). The POMC (panel 1a) and CART (panel 1b) expressions presented an increase of 47% (*p* = 0.003) and 34% (*p* < 0.001), respectively. However, no differences were observed in the orexigenic effectors NPY (*p* = 0.735; panel 1c) and AgRP (*p* = 0.608; panel 1d) mRNA levels.

### 3.3. mRNA Quantification of the Serotonin and Leptin Receptors in the Hypothalamus

**Figure 2:**
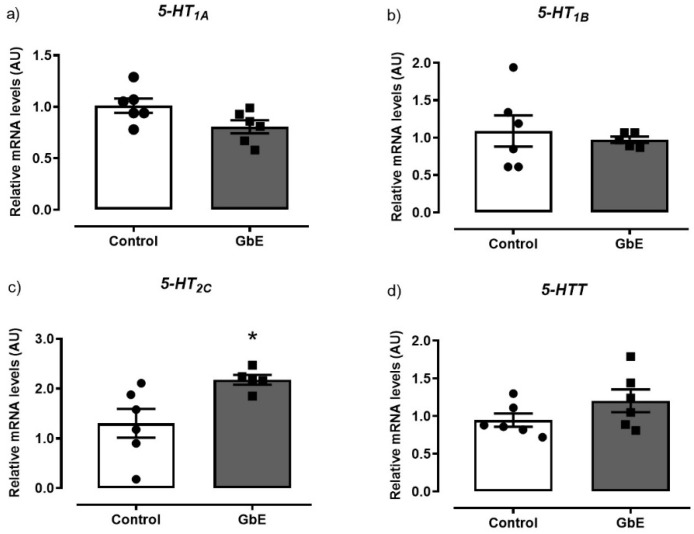
Relative mRNA levels of the serotonin receptors and serotonin transporter: 5-HT1A (**a**), 5-HT1B (**b**), 5-HT2C (**c**), and 5HTT (**d**) of Control (*n* = 5–6) and *Ginkgo biloba* (GbE; *n* = 5–6) groups analyzed by RT-qPCR. * *p* < 0.05.

As it can be seen in Figure 2, GbE supplementation significantly increased mRNA levels of 5-HT2C by 87% (*p* = 0.027; panel 2c). However, no alterations were observed in mRNA expression of neither 5-HT1B (*p* = 0.632; panel 2b,) nor in the 5-HTT (*p* = 0.173; panel 2d). In addition, 5-HT1A mRNA levels (*p* = 0.055; panel 1a) tended to decrease in the GbE group when compared to the Control group.

**Figure 3:**
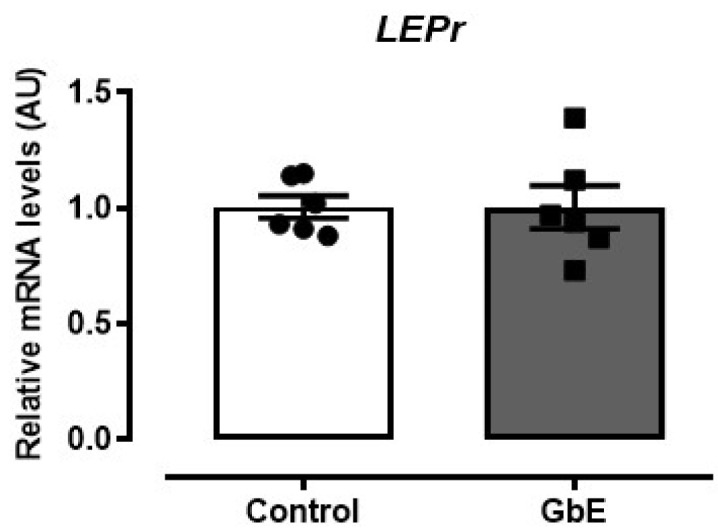
Relative mRNA levels of the leptin receptor (LEPr) in the hypothalamus. Control (*n* = 6) and *Ginkgo biloba* (GbE; *n* = 6) groups analyzed by RT-qPCR.

Furthermore, LepR expression was not altered after the GbE supplementation (*p* = 0.987; Figure 3).

### 3.4. Protein Levels of POMC, 5-HT1B, and 5-HT2C

**Figure 4:**
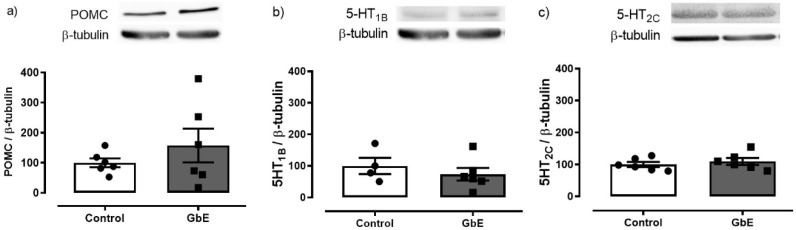
Hypothalamic protein expression. POMC (**a**), 5-HT1B (**b**), and 5-HT2C (**c**) of control (*n* = 4–6) and *Ginkgo biloba* (GbE; *n* = 6) groups.

Protein levels of POMC, 5-HT1B, and 5-HT2C are depicted in Figure 4. A single acute administration of GbE failed to modify the hypothalamic levels of POMC (*p* = 0.818), 5-HT1B (*p* = 0.436), or 5-HT2C (*p* = 0.486).

## 4. Discussion

In the current study, we aimed to evaluate the potential earliest GbE effects on pivotal anorexigenic and orexigenic pathways in the hypothalamus of male rats. Present findings reveal that the administration of a single oral dose of GbE was able to increase the gene expression of anorexigenic effectors, such as the 5-HT2C serotonin receptor and both the POMC and CART neuropeptides, while no changes were observed in the orexigenic effectors. In addition, a declining tendency for hypothalamic 5-HT1A mRNA levels was observed in the GbE group. Thus, present data suggest that the anorexigenic effectors modulation might be one of the first responses involved in the previously reported GbE hypophagic effect [19].

Since no alterations were observed in the protein levels of POMC, 5-HT1B, and 5-HT2C, neither in food intake nor in body mass, we hypothesize that the GbE hypophagic activity might occur after a prolonged GbE supplementation. Indeed, this hypothesis is aligned with previous studies from our group in which the GbE 14-day supplementation reduced food intake, body weight gain, and visceral adiposity in DIO rats [15,16,18]. We have also reported that GbE modulated hypothalamic and hippocampal anorexigenic mechanisms after a 14-day treatment protocol, supporting a hypothesis for a time-dependent hypophagic effect [17,18,19,20,21]. That the GbE effect is time-dependent was also suggested in studies evaluating the modulation of memory. Oliveira and colleagues [22] reported that short-term supplementation with GbE for 1 or 7 days failed to modulate fear memory, while the GbE long-term supplementation for 28 days significantly enhanced it [23].

**Figure 5:**
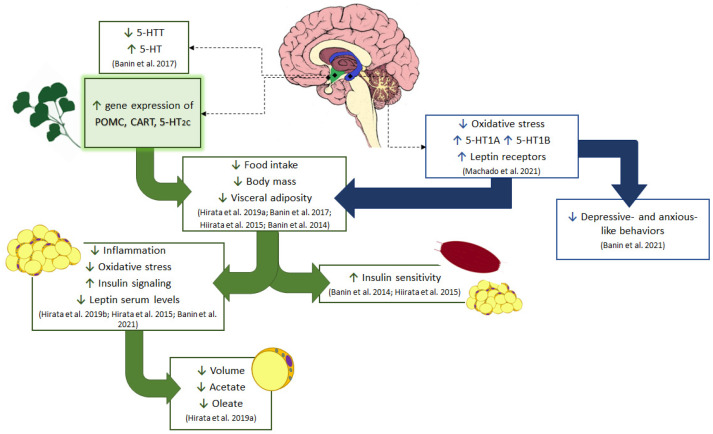
Schematic diagram of the effect of GbE on energy homeostasis. Current results show the GbE effects on hypothalamic anorexigenic pathways, such as increasing the gene expression of 5-HT2C receptor and POMC/CART neuropeptides in normal male rats. The modulation of serotonergic pathways by GbE was also observed in ovariectomized rats with the reduction of the hypothalamic serotonin transporter (5-HTT), restoring the extracellular serotonin (5-HT) levels in the ventromedial hypothalamus as well as re-establishing the hippocampal levels of 5-HT1A and 5-HT1B serotonin receptors. This hippocampal activity together with the decrease in oxidative stress is likely involved in the improvement of depressive- and anxious-like behaviors observed in ovariectomized rats. Additionally, all these central modulations of GbE might have led to the reduction of food intake and body mass in both ovariectomized rats and diet-induced obese male rats. GbE hippocampal activity increased the leptin receptor levels, which participate in the negative feedback of feeding behavior and thus may be involved with the GbE hypophagic effect. Moreover, the GbE central effects might have also favored other responses previously described in diet-induced obese rats, such as the regulation of proteins involved in lipid metabolism, inflammation, and oxidative stress in the retroperitoneal fat depot, as well as the increase of insulin signaling and sensitivity, observed in both the white adipose tissue and gastrocnemius muscle. GbE also reduced the visceral adiposity with a decrease of adipocyte volume, synthesis, and incorporation of fatty acid into triacylglycerol in epididymal adipose tissue.

Souza and colleagues [24] observed that disruptions in POMC expression precede hypothalamic inflammation in obese mice, depicting the important role of POMC neurons on energy homeostasis regulation. However, POMC is a hypothalamic neuropeptide involved in other responses rather than feeding regulation. Alsina and colleagues [25] demonstrated in POMC-deficient mice a diabetogenic phenotype which was reversed by the POMC reactivation, independently of POMC role in energy homeostasis. In addition, enhancement of POMC expression was reported to diminish food intake, body weight gain, visceral adiposity, and the serum levels of cholesterol, insulin, and leptin in genetically obese Zucker rats [26]. It is possible that the beneficial effects of GbE on glucose and cholesterol metabolism, insulin signaling, visceral adiposity, and energy homeostasis previously observed in both the DIO [15,16] and ovariectomized rats [20] might be related to the early modulation of the POMC pathway (Figure 5).

Similarly to the POMC effects, the neuropeptide CART has been shown to reduce food intake, body weight gain, and plasma insulin/leptin levels [27], as well as to increase insulin secretion and decrease glucagon release [28]. On the other hand, disruptions in CART neurons activity also favor the body mass expansion [29] and the appearance of dysfunctions in pancreatic β-cells, which leads to a decrement of insulin secretion [30].

Therefore, considering these findings from the literature aligned with the present results, it is possible to suggest that the modulation of POMC/CART gene expression, as observed after the administration of *Ginkgo biloba* extract, might represent a potential to restore the impaired energy homeostasis associated with obesity.

Although the role of POMC neurons has been highlighted as an important target to the control of feeding behavior and energy homeostasis, the regulation of other anorexigenic pathways may also lead to a remarkable hypophagic response. Nonetheless, serotonergic neurotransmission might be considered as one of the most relevant hypophagic mechanisms [31]. The involvement of the serotonergic system with the food intake control is mainly related to the activation of 5-HT1B and 5-HT2C receptors that respectively inhibits NPY/AgRP signaling and stimulates the POMC pathway [32]. Indeed, the marked hypophagic action promoted by the use of selective serotonin reuptake inhibitors (SSRIs) has been reported to be related to the hypothalamic stimulation of POMC/CART expression [33], depicting the relationship between both pathways.

In the current study, we observed a modulatory effect of GbE on the serotonergic system, since a single dose significantly increased 5-HT2C mRNA and tended to reduce 5-HT1A mRNA, while it did not alter neither the 5-HT1B receptors and serotonin transporter (5-HTT) mRNA levels. Since the activation of the 5-HT2C serotonin receptor stimulates POMC neurons [8], an early increase in 5HT2C gene expression might enhance the responsiveness to 5HT, resulting in a potentiation of the POMC system and finally an anorexigenic response. In addition, 5-HT1A hypothalamic heteroreceptors were reported to be co-localized with NPY and AgRP orexigenic neurons, allowing the authors to suggest that its activation might induce feeding through the release of orexigenic effectors [11]. Despite no statistical difference being observed in 5-HT1A hypothalamic levels, a strong tendency to decline was observed in the GbE group, which might explain, at least in part, why the mRNA levels of orexigenic effectors herein evaluated were not altered. Taken together with previous data showing a stimulatory effect of GbE on the serotonergic system, it is possible to suggest that the activation of anorexigenic pathways precedes the hypophagic effect of GbE.

The present data are particularly interesting when considering the depolarization of POMC/CART neurons via 5-HT2C receptors and hyperpolarization of NPY/AgRP neurons via 5-HT1B receptors in the arcuate hypothalamic nucleus (ARC) under serotonin modulation, which stimulates the downstream of corticotropin-release hormone (CRH) neurons expressed within the paraventricular hypothalamic nucleus (PVH) via activation of MC4R (POMC receptors), and thus promoting satiety [34].

It is important to consider that the therapeutic potential of GbE has been regarded to its unique chemical composition [35]. Indeed, studying ischemia-reperfusion-injured mice, Mu and colleagues [36] proposed that the neuroprotective action of *Ginkgo biloba* is potentiated by the synergic effect of the mixture of the main GbE compounds, the flavonoids, and terpenoids fractions. However, several studies have also shown the metabolic effects of GbE isolated fractions. Both the ginkgolide C and bilobalides were described to modulate proteins involved in lipolysis and adipogenesis in the 3T3-L1 adipose cell line [37,38]. A similar anti-adipogenic effect was observed in the same cell line with the bi-flavone ginkgetin [39], while the improvement of brain metabolism was observed after the treatment with ginkgolide B [40].

It is also important to mention that present data are particularly interesting considering the potential use of GbE as an alternative to treat obesity and its related disorders. Besides the inhibitory effects on food intake, GbE also modulated pivotal proteins involved in lipid metabolism, inflammation, and oxidative stress [17,18]. Remarkably, GbE reduced the adipocyte hypertrophy and the uptake of acetate and oleate in the epididymal fat depot, indicating a potential role of GbE on adipose tissue remodeling as well as on both the synthesis and incorporation of fatty acid into triacylglycerol [17]. Additionally, GbE protected against obesity-related insulin intolerance [15,16]. Considering that the inhibition of anorexigenic signaling pathways favors the establishment of an obesogenic profile [2], the stimulatory effects of GbE supplementation on the gene expression of anorexigenic effectors suggest that the anti-obesogenic effects of GbE previously observed in obese rats might have occurred through the activation of anorexigenic pathways rather than an effect on the orexigenic effectors. Taken together, previous and present findings indicate important central and peripheral effects of GbE, suggesting a potential therapeutic alternative to manage energy homeostasis disturbances.

## 5. Conclusions

In summary, one single acute administration of GbE stimulated the gene expression of anorexigenic effectors, namely POMC, CART, and 5-HT2C receptor, without affecting the mRNA levels of orexigenic neuropeptides NPY/AgRP or leptin receptor. To our knowledge, the current study is the first to demonstrate that the earliest effects of *Ginkgo biloba* on energy homeostasis involve the hypothalamic activation of both the serotonergic system and POMC/CART anorexigenic pathways, shedding a light on the mechanisms of action that precede the hypophagic effect of GbE. However, additional studies are necessary to elucidate the GbE effects on the central signaling pathways involved in the control of energy homeostasis and feeding behavior.

## Figures and Tables

**Table 1:** Food intake (g/100g). *n* = 4–6/group.

Food Intake (g/100g)							
After 1° Saline Gavage				After 2° Saline or GbE Gavage			
Time	Control	GbE	*p* Value	Time	Control	GbE	*p* Value
1 h	0.32 ± 0.10	0.97 ± 0.42	*p* = 0.161	1 h	0.40 ± 0.06	0.24 ± 0.07	*p* = 0.088
4 h	0.42 ± 0.09	0.35 ± 0.09	*p* = 0.588	4 h	0.55 ± 0.09	0.32 ± 0.09	*p* = 0.099
12 h	1.85 ± 0.16	2.08 ± 0.18	*p* = 0.381	12 h	2.56 ± 0.16	2.95 ± 0.23	*p* = 0.192
24 h	4.76 ± 0.24	4.79 ± 0.25	*p* = 0.915	24 h	4.99 ± 0.10	5.31 ± 0.16	*p* = 0.127

**Table 2:** Body weight (g). *n* = 4–6/group.

Body Weight (g)			
Time	Control	GbE	*p* Value
0 h	498.93 ± 4.50	496.80 ± 10.08	*p* = 0.850
24 h	497.99 ± 4.95	496.44 ± 9.55	*p* = 0.888

## Data Availability

All data generated or analyzed in the study will be available in the repository as well as by the corresponding author upon request.

## References

[1] Timper K., Brüning J.C. (2017). Hypothalamic circuits regulating appetite and energy homeostasis: Pathways to obesity. DMM Dis. Model. Mech..

[2] Arora S. (2006). Role of neuropeptides in appetite regulation and obesity—A review. Neuropeptides.

[3] Crespo C.S., Cachero A.P., Jiménez L.P., Barrios V., Ferreiro E.A. (2014). Peptides and food intake. Front. Endocrinol..

[4] Rabe K., Lehrke M., Parhofer K.G., Broedl U.C. (2008). Adipokines and Insulin Resistance. Mol. Med..

[5] Seoane-Collazo P., Fernø J., Gonzalez F. (2015). Hypothalamic-autonomic control of energy homeostasis. Endocrine.

[6] Telles M.M., Guimarães R.B., Ribeiro E.B. (2003). Effect of leptin on the acute feeding-induced hypothalamic serotonergic stimulation in normal rats. Regul. Pept..

[7] Mori R.C.T., Guimarães R.B., Nascimento C.M.O., Ribeiro E.B. (1999). Lateral hypothalamic serotonergic responsiveness to food intake in rat obesity as measured by microdialysis. Can. J. Physiol. Pharmacol..

[8] Heisler L.K., Cowley M.A., Kishi T., Tecott L.H., Fan W., Low M.J., Smart J.L., Rubinstein M., Tatro J.B., Zigman J.M. (2003). Central Serotonin and Melanocortin Pathways Regulating Energy Homeostasis. Ann. N. Y. Acad. Sci..

[9] Voigt J.P., Fink H. (2015). Serotonin controlling feeding and satiety. Behav. Brain Res..

[10] Heisler L.K., Jobst E.E., Sutton G.M., Zhou L., Borok E., Thornton-Jones Z., Liu H.Y., Zigman J.M., Balthasar N., Kishi T. (2006). Serotonin Reciprocally Regulates Melanocortin Neurons to Modulate Food Intake. Neuron.

[11] Collin M., Bäckberg M., Önnestam K., Meister B. (2002). 5-HT1A receptor immunoreactivity in hypothalamic neurons involved in body weight control. Neuroreport.

[12] Donovan M.H., Tecott L.H. (2013). Serotonin and the regulation of mammalian energy balance. Front. Neurosci..

[13] Coulter A.A., Rebello C.J., Greenway F.L. (2018). Centrally Acting Agents for Obesity: Past, Present, and Future. Drugs.

[14] Grass-Kapanke B., Busmane A., Lasmanis A., Hoerr R., Kaschel R. (2011). Effects of Ginkgo Biloba Special Extract EGb 761 in Very Mild Cognitive Impairment (vMCI). Neurosci. Med..

[15] Banin R.M., Hirata B.K.S., Andrade I.S., Zemdegs J.C.S., Clemente A.P.G., Dornellas A.P.S., Boldarine V.T., Estadella D., Albuquerque K.T., Oyama L.M. (2014). Beneficial effects of Ginkgo biloba extract on insulin signaling cascade, dyslipidemia, and body adiposity of diet-induced obese rats. Braz. J. Med. Biol. Res..

[16] Hirata B.K.S., Banin R.M., Dornellas A.P.S., De Andrade I.S., Zemdegs J.C.S., Caperuto L.C., Oyama L.M., Ribeiro E.B., Telles M.M. (2015). Ginkgo biloba extract improves insulin signaling and attenuates inflammation in retroperitoneal adipose tissue depot of obese rats. Mediat. Inflamm..

[17] Hirata B.K.S., Cruz M.M., De Sá R.D.C.C., Farias T.S.M., Machado M.M.F., Bueno A.A., Alonso-Vale M.I.C., Telles M.M. (2019). Potential anti-obesogenic effects of Ginkgo biloba observed in epididymal white adipose tissue of obese rats. Front. Endocrinol..

[18] Hirata B.K.S., Pedroso A.P., Machado M.M.F., Neto N.I.P., Perestrelo B.O., De Sá R.D.C.C., Alonso-Vale M.I.C., Nogueira F.N., Oyama L.M., Ribeiro E.B. (2019). Ginkgo biloba extract modulates the retroperitoneal fat depot proteome and reduces oxidative stress in diet-induced obese rats. Front. Pharmacol..

[19] Banin R.M., de Andrade I.S., Cerutti S.M., Oyama L.M., Telles M.M., Ribeiro E.B. (2017). Ginkgo biloba Extract (GbE) stimulates the hypothalamic serotonergic system and attenuates obesity in ovariectomized rats. Front. Pharmacol..

[20] Banin R.M., Machado M.M.F., de Andrade I.S., Carvalho L.O.T., Hirata B.K.S., de Andrade H.M., da Silva Júlio V., Ribeiro J.d.S.F.B., Cerutti S.M., Oyama L.M. (2021). Ginkgo biloba extract (GbE) attenuates obesity and anxious/depressive-like behaviours induced by ovariectomy. Sci. Rep..

[21] Machado M.M.F., Banin R.M., Thomaz F.M., de Andrade I.S., Boldarine V.T., Figueiredo J.d.S., Hirata B.K.S., Oyama L.M., Lago J.H.G., Ribeiro E.B. (2021). Ginkgo biloba Extract (GbE) Restores Serotonin and Leptin Receptor Levels and Plays an Antioxidative Role in the Hippocampus of Ovariectomized Rats. Mol. Neurobiol..

[22] Oliveira D.R., Sanada P.F., Saragossa F.A.C., Innocenti L.R., Oler G., Cerutti J.M., Cerutti S.M. (2009). Neuromodulatory property of standardized extract Ginkgo biloba L. (EGb 761) on memory: Behavioral and molecular evidence. Brain Res..

[23] Oliveira D.R., Sanada P.F., Filho A.C.S., Conceição G.M.S., Cerutti J.M., Cerutti S.M. (2013). Long-term treatment with standardized extract of Ginkgo biloba L. enhances the conditioned suppression of licking in rats by the modulation of neuronal and glial cell function in the dorsal hippocampus and central amygdala. Neuroscience.

[24] Souza G.F.P., Solon C., Nascimento L.F., De-Lima-Junior J.C., Nogueira G., Moura R., Rocha G.Z., Fioravante M., Bobbo V., Morari J. (2016). Defective regulation of POMC precedes hypothalamic inflammation in diet-induced obesity. Sci. Rep..

[25] Alsina R., Trotta M., Bumaschny V.F. (2018). Hypothalamic Proopiomelanocortin Is Necessary for Normal Glucose Homeostasis in Female Mice. Front. Endocrinol..

[26] Li G., Mobbs C.V., Scarpace P.J. (2003). Central pro-opiomelanocortin gene delivery results in hypophagia, reduced visceral adiposity, and improved insulin sensitivity in genetically obese Zucker rats. Diabetes.

[27] Rohner-Jeanrenaud F., Craft L.S., Bridwell J., Suter T.M., Tinsley F.C., Smiley D.L., Burkhart D.R., Statnick M.A., Heiman M.L., Ravussin E. (2002). Chronic central infusion of cocaine- and amphetamine-regulated transcript (CART 55-102): Effects on body weight homeostasis in lean and high-fat-fed obese rats. Int. J. Obes..

[28] Abels M., Riva M., Bennet H., Ahlqvist E., Dyachok O., Nagaraj V., Shcherbina L., Fred R.G., Poon W., Sörhede-Winzell M. (2016). CART is overexpressed in human type 2 diabetic islets and inhibits glucagon secretion and increases insulin secretion. Diabetologia.

[29] Bartell S.M., Isales C.M., Baile C.A., Kuhar M.J., Hamrick M.W. (2008). CART deficiency increases body weight but does not alter bone strength. J. Musculoskelet. Neuronal Interact..

[30] Wierup N., Richards W.G., Bannon A.W., Kuhar M.J., Ahrén B., Sundler F. (2005). CART knock out mice have impaired insulin secretion and glucose intolerance, altered beta cell morphology and increased body weight. Regul. Pept..

[31] Wyler S.C., Lord C.C., Lee S., Elmquist J.K., Liu C. (2017). Serotonergic control of metabolic homeostasis. Front. Cell. Neurosci..

[32] Feijó F.d.M., Bertoluci M.C., Reis C. (2011). Serotonin and hypothalamic control of hunger: A review. Rev. Assoc. Med. Bras..

[33] Nonogaki K., Kaji T. (2016). The acute anorexic effect of liraglutide, a GLP-1 receptor agonist, does not require functional leptin receptor, serotonin, and hypothalamic POMC and CART activities in mice. Diabetes Res. Clin. Pract..

[34] Garfield A.S., Heisler L.K. (2009). Pharmacological targeting of the serotonergic system for the treatment of obesity. J. Physiol..

[35] Heinonen T., Gaus W. (2015). Cross matching observations on toxicological and clinical data for the assessment of tolerability and safety of Ginkgo biloba leaf extract. Toxicology.

[36] Mu L., Kou J., Zhu D., Yu B. (2007). Comparison of neuroprotective effects of flavonoids, terpenoids, and their combinations from Ginkgo biloba on ischemia-reperfusion—Injured mice. Pharm. Biol..

[37] Bu S., Yuan C.Y., Xue Q., Chen Y., Cao F. (2019). Bilobalide suppresses adipogenesis in 3T3-L1 adipocytes via the AMPK signaling pathway. Molecules.

[38] Liou C.J., Lai X.Y., Chen Y.L., Wang C.L., Wei C.H., Huang W.C. (2015). Ginkgolide C suppresses adipogenesis in 3T3-L1 adipocytes via the AMPK signaling pathway. Evidence-Based Complement. Altern. Med..

[39] Cho Y.L., Park J.G., Kang H.J., Kim W., Cho M.J., Jang J.H., Kwon M.G., Kim S., Lee S.H., Lee J. (2019). Ginkgetin, a biflavone from Ginkgo biloba leaves, prevents adipogenesis through STAT5-mediated PPARγ and C/EBPα regulation. Pharmacol. Res..

[40] Chi C.-L., Shen D.-F., Wang P.-J., Li H.-L., Zhang L. (2015). Effect of ginkgolide B on brain metabolism and tissue oxygenation in severe haemorrhagic stroke. Int. J. Clin. Exp. Med..

